# Exposure to Different Amounts of Dietary Gluten in Patients with Non-Celiac Gluten Sensitivity (NCGS): An Exploratory Study

**DOI:** 10.3390/nu11010136

**Published:** 2019-01-10

**Authors:** Leda Roncoroni, Karla A. Bascuñán, Maurizio Vecchi, Luisa Doneda, Maria T. Bardella, Vincenza Lombardo, Alice Scricciolo, Federica Branchi, Luca Elli

**Affiliations:** 1 Center for Prevention and Diagnosis of Celiac Disease, Gastroenterology and Endoscopy Unit, Fondazione IRCCS Ca’ Granda Ospedale Maggiore Policlinico, 20135 Milan, Italy; mariateresa.bardella@yahoo.com (M.T.B.); vicky.l@hotmail.it (V.L.); scricciolo.alice@gmail.com (A.S.); federica.branchi@gmail.com (F.B.); dottorlucaelli@gmail.com (L.E.); 2 Department of Biomedical, Surgical and Dental Sciences, University of Milan, 20133 Milan, Italy; luisa.doneda@unimi.it; 3 Department of Nutrition, School of Medicine, University of Chile, 8380453 Santiago, Chile; 4 Department of Pathophysiology and Transplantation, University of Milan, 20122 Milan, Italy; maurizio.vecchi@policlinico.mi.it; 5 Gastroenterology and Endoscopic Unit, Fondazione IRCCS Ca’ Granda, Ospedale Maggiore Policlinico, 20122 Milan, Italy

**Keywords:** non-celiac gluten sensitivity, gluten re-introduction, gluten-free diet, gastrointestinal symptoms

## Abstract

It is unclear whether patients with non-celiac gluten sensitivity (NCGS) can tolerate gluten. We have evaluated the changes of both gastrointestinal symptoms and quality of life for NCGS patients after the re-introduction of dietary gluten. Twenty-two NCGS patients reporting functional gastroenterological symptoms and on gluten-free diet (GFD) for the previous three weeks were exposed to incremental gluten-containing diets. Three groups were compared at baseline (immediately after 3-weeks on GFD) and immediately after the return of symptomatology: (i) a group tolerating a low-gluten diet (3.5 g gluten/day, week 1, *n* = 8), (ii) a group tolerating a mid-gluten diet (8 g gluten/day, week 2, *n* = 6), and (iii) a group tolerating a high-gluten diet (13 g gluten/day, week 3, *n* = 8). Their gastrointestinal symptoms and quality of life were assessed at baseline and post-intervention. The most common symptoms were: constipation (46%), abdominal pain (50%) and dyspepsia (38%). A decrease in several short form health survey (SF-36) sub-scores (all *p* < 0.03) after gluten re-introduction was only observed in the group tolerating the low-gluten diet; the same group showed a lower post-intervention role-emotional SF-36 score (*p* = 0.01). Most gastrointestinal symptoms remained similar after gluten re-introduction. However, a decrease in the general perception of well-being was only found after gluten re-introduction in the group tolerating a low-gluten diet (*p* = 0.01); the same was true when comparing the post-intervention general well-being perception among the three groups (*p* = 0.050). In conclusion, dissimilar responses from patients with NCGS were observed after the re-introduction of gluten, with gluten at a low dosage affecting the quality of life and general well-being of a group of patients, whereas others tolerate even higher doses of dietary gluten.

## 1. Introduction

Non-celiac gluten sensitivity (NCGS) is characterized by intestinal and extra-intestinal symptoms related to the ingestion of gluten-containing foods, in subjects that are not affected by either celiac disease (CD) or wheat allergy [1,2,3]. The symptomatology commonly found in NCGS comprises: bloating, abdominal pain, diarrhea, epigastric pain, nausea, aerophagia, lack of well-being, tiredness, headache, foggy mind, and anxiety among other symptoms [4]. Symptoms disappear after starting on a gluten-free diet (GFD) and appear again after a gluten challenge within a few hours or a couple of days [5,6]. However, this latter finding can be attributed to a placebo/nocebo effect [7,8]. Several studies have evaluated the effect of a gluten re-challenge in NCGS patients after GFD (a summary of studies is shown in Table 1). According to a recent meta-analysis, there is a wide range of patients relapsing after a gluten challenge (between 7% and 77%) and no effect of a gluten challenge was found on the risk of relapse [9]. These results are in line with another review of studies on patients with suspected NCGS, indicating that only 16% of them show clear gluten-specific symptoms [10]. These studies highlight the fact that further methodological considerations are required in studies evaluating the gluten challenge. 

The current clinical consensus is that the diagnostic criteria on NCGS should include self-reported gluten intolerance, negative serology for CD (including immunoglobulin A (IgA) endomysial antibodies, IgA tissue transglutaminase antibodies, and IgG de-amidated gliadin peptide antibodies) and the absence of villous atrophy at duodenal histology (whilst on a gluten-containing diet) [1,3,11]. 

Similarly to CD and wheat allergy, the cornerstone of NCGS treatment is the withdrawal of gluten-containing foods. Although considered safe and effective, the lifelong elimination of gluten from the diet carries psychological and social implications. Patients with CD report about concerns related to the management of their social relationships and life routine [12]. Support and education are important to enable patients to adapt to their new diet [13]. However, given the uncertainty on the pathogenesis and trigger(s) of NCGS, it is not clear how strict such a new diet needs to be, how long its implementation and how to monitor the efficacy of the treatment other than by clinical response. Clinical experience suggests that patients affected by NCGS range from those who need to adhere to a strict GFD to those who can tolerate potential cross-contamination without any clinical consequences [14].

NCGS is a disorder treated with a GFD. There is currently discussion whether the symptoms described in NCGS are exclusively due to the ingestion of gluten proteins rather than other components included in wheat [15]. Wheat has some components that are different from gluten proteins and can be harmful to patients suffering from NCGS, including wheat germ agglutinins (WGA), amylase inhibitors/trypsin (ATI), and fermentable oligo/di/monosaccharides and polyols (FODMAP) [16,17,18,19]. ATIs are a family of structurally similar proteins, which serve as protective proteins in wheat and other cereals, by inhibiting the enzymes (trypsin and trypsin-like activities) of wheat and some parasites [20] ATIs have been described as triggers of the activation of innate immunity in intestinal cells [18]. WGAs [19], similar to ATIs, serve as protective proteins as they are resistant to heat and proteolysis. WGAs have shown to promote the production of pro-inflammatory cytokines, which affect the integrity of the intestinal epithelium [21]. Finally, FODMAP-containing foods include such components as oligosaccharides, disaccharides, monosaccharides, and sugar alcohols. They are resistant to digestion and can ferment completely or partially in the large intestine. Their efficacy in the treatment of gastrointestinal symptoms related to IBS has been described, and their function is being evaluated in various pathologies affecting the intestine [22,23].

There is currently no data that can support any recommendations on the need for, or frequency of, repeated follow-up visits in these patients. It is considered good clinical care to study these patients at regular intervals in order to ensure they remain healthy and to involve a nutritionist to make sure they are not at risk of nutrient deficiencies. It is also advisable that the continued need for “strict” avoidance of all gluten-related products be regularly reviewed following recovery because some patients can possibly follow a less restrictive diet with no recurrence of symptoms. A lifelong strict GFD (as in CD) vs. an “on-demand” approach is the main question. Many experts recommend that patients should undergo periodic re-evaluation with the re-introduction of gluten (e.g., every 6–12 months) [8]. 

A GFD leads to the complete disappearance of symptoms in most patients with NCGS but in some cases the level of improvement after gluten withdrawal is only partial. However, it should be mentioned that the level of tolerance varies among individuals and there are patients with NCGS who do not tolerate even very small amounts of gluten. As it is presently unclear whether gluten sensitivity is a permanent or transient condition, the re-introduction of gluten after 1–2 years on a GFD is potentially advisable [33]. Thus, the aim of the present study was to evaluate the changes in gastrointestinal symptoms and quality of life for NCGS patients after exposure to different amounts of dietary gluten.

## 2. Materials and Methods

Between 2013 and 2014, patients reporting functional gastroenterological symptoms according to the Rome III criteria [34] were invited to participate in this study. All were recruited from the gastroenterological outpatient clinic at the Center for Prevention and Diagnosis of Celiac Disease, Gastroenterology and Endoscopy Unit, Fondazione IRCCS Ca’ Granda Ospedale Maggiore Policlinico in Milan (Italy). The patients who agreed to participate gave their written informed consent and were enrolled in the study. The local Ethics Committee for Human Research of the City of Milan approved the study protocol. The trial was registered in ClinicalTrial.gov (NCT01864993).

The inclusion criteria were: adult age (>18 years old), being on a gluten-containing diet, with negative anti-tissue transglutaminase IgA, normal IgA dosage, negative IgE-mediated wheat allergy as verified by skin prick test and serological IgE dosage. The exclusion criteria were: diagnosis of CD, wheat allergy, inflammatory bowel disease, psychiatric disorders, major abdominal surgery (in particular, intestinal resections), diabetes mellitus, systemic autoimmune diseases, previous anaphylactic episodes, any systemic disorders, patients already following or having followed a GFD regimen in the previous six months, pregnant or breastfeeding women, and patients already on pharmacological therapy. The patients were evaluated by a gastroenterologist and a qualified nutritionist. The diagnosis of NCGS was made in accordance with the latest NCGS consensus [4]. After recruitment, patients were requested to follow a GFD plan for 3-weeks before the start of the dietary intervention (i.e., the low/mid/high-gluten diet). Their overall health, gastrointestinal symptoms, and quality of life were assessed by medical examination. Their adherence to the GFD was evaluated according to the celiac dietary adherence test (CDAT) [35]. The CDAT is a clinically relevant, easily administrated 7-item instrument which allows the standardized evaluation of GFD adherence. It is a sensitive tool developed using standard psychometric techniques. Only those patients with excellent or very good GFD adherence were included in the study. CDAT is based on a score ranging from 7 to 35 against seven questions, each on a 5-point scale, with higher scores denoting worse GFD adherence [35].

### 2.1. Intervention

**Figure 1:**
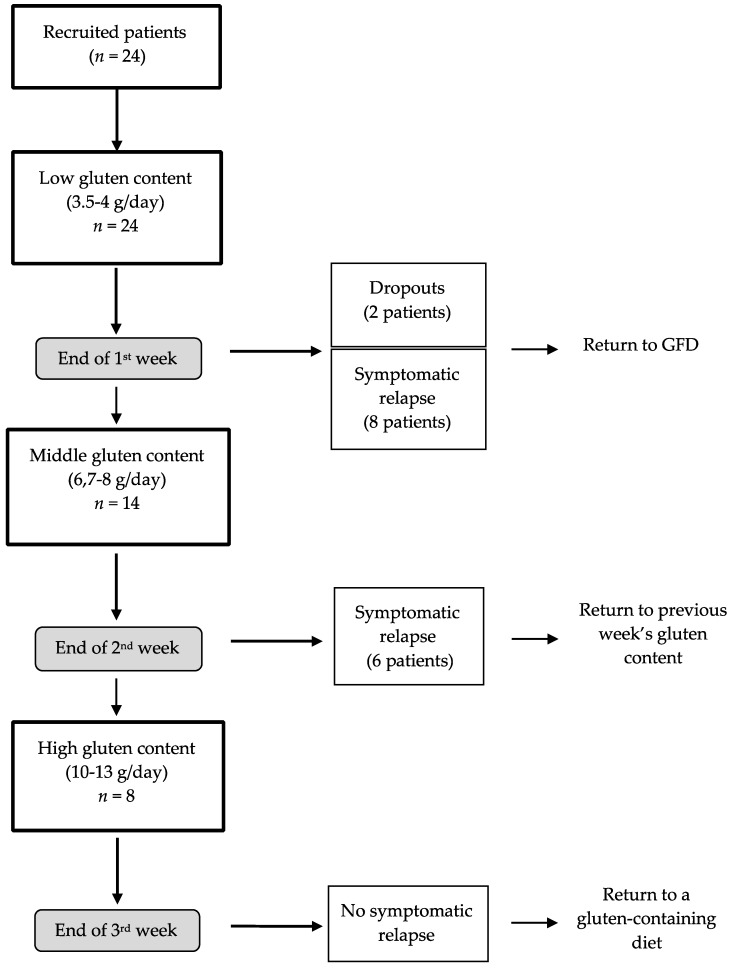
Flow-chart of the patients’ activity in the study. Two patients (drop-outs) decided to abandon the diet during the first week of intervention; GFD: gluten-free diet.

Twenty-four patients were recruited (Figure 1). As mentioned above, all the recruited patients were instructed to follow a strict GFD for 3 weeks. After that time, the intervention period started. A qualified nutritionist designed a personalized GFD adjusted to match daily requirements for energy, macronutrients and micronutrients. A structured 3-week dietary plan was indicated and explained to every patient, to cover structured meals, foods/beverages and alternatives for food purchase. The patients were also encouraged to immediately contact the nutritionist by phone in case of any doubt related to the diet. After the three weeks on the GFD, the intervention period started. The patients started the study with a low-gluten diet during the first week (3.5–4 g gluten/day, week 1, *n* = 24). Two patients dropped out of the study during the first week because they did not want to continue the diet. Afterwards, the patients who did not report adverse symptoms were administered a mid-gluten diet in the second week (6.7–8 g gluten/day, week 2, *n* = 14). Then, the patients who passed the second week were started on a high-gluten diet for the following week (10–13 g gluten/day, week 3, *n* = 8). Each patient had been instructed to immediately contact the research team at the end of each week should any of the previously reported symptoms at the beginning of the study return. A clinical evaluation was then arranged and the patient was to stop their gluten-containing diet and return to the GFD (i.e., for patients reporting adverse symptomatology at the end of the first week) or to the previous gluten-containing diet (i.e., for patients reporting adverse symptomatology at the end of the second and third week). In such cases, the nutritionist would also reinforce the instructions and food education on the practice of the GFD. A flow-chart of patients is shown in Figure 1. The patients with symptomatic relapse at the end of week 1 returned to the GFD (as indicated in the previous three weeks after recruitment). The patients who experienced a symptomatic relapse at the end of week 2 returned to a low-gluten diet (3.5–4 g/day) and stayed on that dietary treatment until the end of week 3. Finally, none of the patients who had undergone the high-gluten diet (*n* = 8) reported any worsening of gastrointestinal symptoms and at the end of week 3, they were instructed to return to their regular dietary pattern.

### 2.2. Diets

The nutritional evaluation aimed to assess anthropometrical parameters, nutritional status, and usual dietary patterns. At the beginning of the study, after clinical evaluation, a structured 7-day dietary plan was generated for each patient, adjusted to his/her daily nutritional requirements for energy, macronutrients and micronutrients. For each week, according to the low/mid/high-gluten amount contained, meals were listed (breakfast, morning snack, lunch, afternoon snack, dinner and other snacks during the day) with specific foods/beverages (see examples in Table 2). For week 1 the source of gluten was only wheat pasta (50 g, about 3.5–4 g of gluten) administered during dinner. In week 2 the sources of gluten were wheat pasta (50 g, about 3.5–4 g of gluten) during dinner and wheat bread (50 g, about 3.2–4 g gluten) during the daytime. For week 3 the sources of gluten were wheat pasta (60 g at lunch and 60 g at dinner, ~8.4–9.6 g gluten) and wheat bread (30 g during the day, 1.9–3 g gluten). The gluten content of each of the three diets was calculated referring to Schalk et al. [36]. In that study, the gluten content was determined through a comprehensive strategy to isolate gluten protein fractions and gluten protein types (GPT) from wheat, rye, barley, and oat flours. All of the isolated GPTs were fully characterized by means of analytical reversed-phase high-performance liquid chromatography (RP-HPLC), sodium dodecyl sulfate-polyacrylamide gel electrophoresis (SDS-PAGE), N-terminal sequencing, electrospray-ionization quadrupole time-of-flight mass spectrometry (LC-ESI-QTOF-MS) and untargeted LC-MS/MS of chymotryptic hydrolysates of the single GPT. Successively, all of the GPTs were reproducibly isolated in high purity from the flours and were made suitable to be used as a reference material, i.e., for calibration of liquid chromatography tandem mass spectrometry methods or enzyme-linked immunosorbent assays (ELISAs) [36].

### 2.3. Gastrointestinal Symptoms and Quality of Life

The gastrointestinal symptoms and quality of life of each patient were assessed at the beginning of the study and soon after the return of symptoms after administering one of the gluten-containing diets (i.e., after gluten exposure). A visual analogue scale (VAS) was used to assess the patient’s gastrointestinal symptoms and general perception of well-being as previously described by our group [23]. This instrument recorded the severity of specific symptoms: abdominal pain, bloating, postprandial fullness, early satiety, epigastric pain, non-specific functional gastrointestinal symptoms, and satisfaction with stool consistency. For each question, each patient was asked to put a mark along a 10-cm long line with one end 0 meaning “absence of symptom” and the other end 10 “severe symptomatology”. A further VAS evaluated the satisfaction about the current level of general well-being, with 0 meaning “completely unsatisfied” and 10 “absolutely satisfied”.

The patient’s quality of life was evaluated through the short form health survey (SF-36) questionnaire. This instrument comprises 36 questions that conceptually refer to eight health domains [37]. The patients were asked to answer each question and then domain-specific scores ranging between 0 and 100 were calculated, where 100 represented the best possible perception of quality of life. 

### 2.4. Statistical Analysis

The data are provided as mean ± SEM or median (interquartile range) unless indicated otherwise. Twenty-two patients were included for analysis. Comparisons were made according to the group of patients that reported the return of symptomatology after gluten exposure, that is 3 groups: low-gluten (patients that reported adverse symptomatology after 1 week on a gluten-containing diet, *n* = 8), mid-gluten (patients that reported adverse symptomatology after 2 weeks on a gluten-containing diet, *n* = 6), and high-gluten (patients that reported adverse symptomatology after 3 weeks on a gluten-containing diet, *n* = 8). One-way ANOVA was used to evaluate between-group differences as to age and body-mass index; Fisher’s exact test was used to compare categorical variables (i.e., gender distribution and presence of gastrointestinal symptoms) between the groups. The within-group differences of SF-36 and VAS scores before (immediately after 3-weeks on a GFD) and after gluten exposure (i.e., baseline vs. the time when gastrointestinal symptoms returned after the gluten-containing diets) were assessed using a non-parametric Wilcoxon’s rank sum test. The between-group differences were evaluated after gluten exposure using the non-parametric Kruskal-Wallis test. STATA^®^ ver. 13.1 software (StataCorp, College Station, TX, USA) was used for statistics and statistical significance was set at a 5% α-level.

## 3. Results

### 3.1. Patients

All patients included in the study obtained a CDAT score from 7 to 13, thus indicating very good adherence to the GFD. As shown in Table 3, the patients were middle-aged, mainly women, and within the normal weight range. Regarding the clinical symptomatology at baseline, symptoms such as constipation, abdominal pain, and dyspepsia were the most frequently reported by the whole sample (46%, 50%, and 38%, respectively). All three groups were comparable regarding both general characteristics and present gastrointestinal symptoms at baseline (Table 3). In regards to the estimated gluten content in the foods used in the intervention diets, the gluten content was 3.5–4 g/day in the low-gluten diet, 6.7–8 g/day in the mid-gluten diet, and 10–13 g/day in the high-gluten diet.

### 3.2. Quality of Life

The resulting SF-36 scores are shown in Table 4. There was a significant decrease in several SF-36 sub-scores (role physical, role emotional, bodily pain, mental health, vitality and social interaction, all *p* < 0.03) after gluten exposure in the group of patients receiving the low-gluten diet but not in the groups receiving mid- and high-gluten content (Table 4). However, when comparing the change in SF-36 scores after dietary gluten exposure between the three groups, we observed a change only in the role emotional score, which was lower in the low-gluten content group. No post-intervention differences were found regarding the general health score among the three groups (Table 4). 

### 3.3. Gastrointestinal Symptoms

The within-group comparisons showed no significant changes in most of the evaluated gastrointestinal symptoms before and after dietary intervention (Table 5). However, a decrease in the general perception of well-being was found in the low-gluten group (but not in the mid- and high-gluten groups) after intervention (*p* = 0.01). In line, when comparing the three groups after dietary gluten exposure, a further decrease of the general well-being level was found in the low-gluten group compared with the mid- and high-gluten groups (*p* = 0.050, Table 5). 

## 4. Discussion

This study evaluated the effects of a short-term re-introduction of gluten on individuals diagnosed with NCGS. Our results show that a level of tolerance is present in patients without showing any adverse signs or gastrointestinal symptoms when consuming gluten. There was a different response among individuals with NCGS when exposed to different amounts of dietary gluten. A subgroup of patients had an immediate response with some worsening of their quality of life and general well-being at a low-gluten dosage, whereas other patients were able to tolerate medium and high doses of gluten, indicating that these latter groups can be administered some gluten without adverse health effects.

At present, it is well known that a GFD is the treatment of choice for patients suffering from NGCS. Several randomized controlled trials (RCTs) [10] have been carried out to identify gluten as the trigger of symptoms (Table 1). Those reports have shown variable results and are not conclusive regarding the cause-effect relationship of gluten and gastrointestinal symptoms [30,32]. We have previously suggested that gluten can be a major trigger of gastrointestinal symptoms in line with other [5,38,39]. Although the data to date suggest a benefit from a GFD for a selected group of patients, it is possible that the improvement in symptoms might not be due to gluten itself. Other components in wheat may trigger the reported symptoms in these patients, suggesting the clinical feature of non-celiac wheat sensitivity. This last entity has not completely been clarified as it is not clear whether patients are suffering from gluten-related symptoms or another component of wheat (such as fructans) [40]. Regardless of the nomenclature, Carroccio et al. [41] provided a clinically useful approach confirming non-celiac wheat sensitivity as a unique clinical condition. Their results suggest the existence of two different groups of patients with this condition: one with characteristics similar to CD and the other with characteristics resembling food allergy [41]. The current nomenclature of gluten sensitivity [1], NCGS [5] and gluten-related disorders does not resolve this problem and may confuse clinicians as to which component in wheat might be triggering patients’ symptomatology. Expert recommendations have proposed a periodic evaluation with re-introduction of gluten for NCGS patients on consideration of the economic costs and quality of life that a lifelong GFD entails [8]. 

Regarding the quality-of-life perception, previous data of our group from a cross-over study has shown that patients with NCGS treated with a GFD enjoy an improvement in the majority of the SF-36 scores after 7 days [30]. In our study, we observed that the group who tolerated only a low amount of dietary gluten was the only group showing a decrease in several SF-36 sub-scores. On the other hand, it is important to point out that the groups on a mid- and high-gluten diet did not show any significant change in their quality of life. This finding is intriguing because it would suggest that a greater gluten intake by patients with NCGS would not necessarily further affect their quality of life, thus reinforcing the idea of inter-individual variability against gluten in this group.

After the re-introduction of gluten, the gastrointestinal symptomatology showed no main changes against our dietary intervention. Moreover, no differences were found after gluten exposure among the three groups (i.e., at the end of the intervention period). However, we did find a change in the perception of general well-being, which was significantly affected in the group receiving a low level of gluten; such a result is in line with what we found on the patients’ quality of life. Overall, these findings suggest that with regard to quality of life and general well-being the changes observed in the group on a low-gluten diet would be related to a systemic response to gluten consumption rather than only gastrointestinal symptomatology or, at least, a combination of both. Even if our results are interesting per se they require further confirmation in larger samples and with different populations of patients suffering from NCGS. 

This was an exploratory study that worked on a small sample of patients to evaluate the re-introduction of gluten through dietary modifications in a homogeneous group of patients correctly diagnosed with NCGS. As to limitations, we would like to point out that all the patients did not receive all their gluten doses in a balanced cross-over design. This was mainly due to ethical considerations since, at the time the clinical picture began to worsen, the patients stopped the administered diet and returned to their established treatment with GFD. 

To summarize, we have shown a dissimilar response after the reintroduction of gluten in patients with NCGS who were on GFD for the last 3-weeks. We have also shown that for a group of them the re-introduction of gluten at low dosage affected their quality of life and general well-being, whereas other patients could tolerate higher doses of dietary gluten. Further studies are needed to establish whether NCGS patients require a dietary regimen free of gluten or just a gluten-restricted diet. Therefore, a controlled re-introduction of gluten potentially helps the improvement of selected patients that are able to tolerate gluten intake by developing a personalized diet containing gluten without the reappearance of symptoms. Further research is needed to assess the long-term clinical response of the increase in the dietary gluten content as concerns symptomatology and quality of life for patients with NCGS. 

## Figures and Tables

**Table 1:** Summary of clinical trials evaluating a gluten re-challenge in NCGS patients.

Study (Reference)	Patients	Study Design	No. of Patients	Challenge Duration	Methods	Main Findings
Biesiekierski et al. [5]	NCGS w/IBS	DBRPCT	34 patients	up to 6 weeks	Gluten or placebo (two bread slices plus one muffin per day)	68% (13) of patients in the gluten group reported inadequate symptoms control compared with 40% (6) under placebo.
Carroccio et al. [24]	NCGS	DBRPCT		2 weeks	Capsules with wheat (20 g) vs. placebo	30% (276) of patients diagnosed as non-celiac wheat sensitivity. Wheat challenge induced >30% increase in symptoms.
Peters et al. [25]	NCGS w/IBS symptoms	DBRPCT	22 patients	3 days	Dietary challenge: gluten-free food supplemented with gluten (16 g/day), whey (16 g/day) or not supplemented (placebo)	Higher overall Spielberger State Trait Personality Inventory state depression scores compared to placebo but not whey, after gluten. Similar gastrointestinal symptoms induced after all treatments
Zanini et al. [26]	NCGS	DBRPCT	35 non-CD patients	10 days	Gluten-containing flour or gluten-free flour for 10 days, followed by a 2-week washout period	34% of patients diagnosed as having NCGS
Capannolo et al. [27]	NCGS	DBRPCT	364 patients (27 with NCGS and 337 with no specific diagnosis)	1 month	Challenge with dietary gluten vs. a GFD.	85.96% did not experience a change in symptomatology after a GFD. Low value of a gluten-containing diet for an increase in symptoms.
Rosinach et al. [28]	NCGS	DBRPCT	18 non-CD patients	6 months	11 gluten (20 g/day) and 7 placeboes	91% of patients with clinical relapse during gluten challenge (vs. 28.5% after placebo)
Picarelli et al. [29]	NCGS	DBRPCT	26 patients	1 day	Food challenge (oral provocation test) with a gluten-containing croissant (10 g of gluten per croissant) to 13 patients and a gluten-free croissant to the other 13 patients.	No difference was found between the severity of symptoms with gluten-containing croissants compared to a group of patients with gluten-free croissants.
Elli et al. [30]	NCGS w/functional gastrointestinal symptoms	DBRPCT	98 patients	7 days	Gluten intake (5.6 g/day) or placebo	28 patients reported symptomatic relapse with deteriorated quality of life
Skodje et al. [31]	Subjects with self-reported NCGS	DBRPCT	59 subjects	7 days	Diets containing gluten (5.7 g), fructans (2.1 g), or placebo	Overall Gastrointestinal Symptom Rating Scale for Irritable Bowel Syndrome scores increased after fructans rather than gluten.
Dale et al. [32]	Patients w/suspected NCGS	DBRPCT	20 patients	4 days	Two muffins a day (11/0 g gluten). (4 periods w/double-blinded provocation, 2 w/gluten, 2 w/placebo)	Most severe symptoms found after placebo. Only 4/20 patients correctly identified periods w/gluten.

NCGS: Non-celiac gluten sensitivity; DBRPCT: Double-blind randomized placebo-controlled trial; IBS: Irritable bowel syndrome; CD: Celiac disease; GFD: Gluten-free diet.

**Table 2:** Example of the three dietary plans used and differing in the amount of gluten contained ^1^.

Meal	Low-Gluten (3.5–4 g Gluten)	Mid-Gluten (6.7–8 g Gluten)	High-Gluten (10–13 g Gluten)
Breakfast	1 small cup of coffee, 300 mL partly skimmed milk, 1 gluten-free croissant	1 small cup of coffee, 300 mL partly skimmed milk, 4 gluten-free biscuits	1 small cup of coffee, 300 mL partly skimmed milk, 4 gluten-free rusks
Morning snack	1 kiwifruit	1 apple	1 banana
Lunch	100 g gluten-free pasta, 90 g mixed vegetables, 1 portion of chard, 4 mandarins	100 g gluten-free pasta, 40 g cow ricotta cheese, 100 g potatoes, 2 bananas	60 g wheat pasta with broccoli, 2 teaspoonfuls grated Parmesan cheese, 1 portion of mixed salad, 2.5 glasses of fruit salad
Afternoon snack	1 bowl of strawberries	1 bowl of strawberries	1 pear
Dinner	50 g wheat pasta with zucchini, 100 g turkey thigh 200 g potatoes, 5 slices of pineapple	50 g wheat pasta with tomato sauce, 1 spoon of fresh peas 120 g pork, 1 portion of chard 5 mandarins	60 g wheat pasta with tomato sauce, 100 g halibut, 200 g potatoes, 2 glasses of fruit salad
During the day	40 g gluten-free bread, 6.5 teaspoonfuls virgin olive oil	50 g of white wheat bread, 3.5 teaspoonfuls virgin olive oil	30 g white wheat bread, 8 teaspoonfuls virgin olive oil

^1^ Example of a typical day per week for each of the three different diets, as calculated for an average 2000 Kcal/day energy requirement.

**Table 3:** General characteristics of the group of patients at baseline ^1^.

	Low-Gluten (3.5–4 g/day, *n* = 8)	Mid-Gluten (6.7–8 g/day, *n* = 6)	High-Gluten (10–13 g/day, *n* = 8)	*p*-Value ^†^
Age, years	44.6 ± 4.5	45.5 ± 3.1	44.6 ± 5.2	0.98
Gender, F/M	7/1	6/0	7/1	0.99
BMI, kg/m^2^	23.0 ± 1.7	23.8 ± 1.8	21.8 ± 0.7	0.65
Diarrhea, *n* (%)	1 (12.5)	0 (0)	1 (12.5)	0.99
Bloating, *n* (%)	0 (0)	2 (33.3)	1 (12.5)	0.24
Constipation, *n* (%)	3 (37.5)	5 (83.3)	3 (37.5)	0.21
Abdominal pain, *n* (%)	5 (62.5)	4 (66.6)	3 (37.5)	0.64
Dyspepsia, *n* (%)	6 (75)	1 (16.6)	2 (25)	0.08

^1^ Data are shown as mean ± SEM for continuous variables and frequency and/or relative number for nominal variables. ^†^ *p*-value for comparison between groups using one-way ANOVA or Fisher’s exact test for categorical variables. F: female; M: male; BMI: body mass index.

**Table 4:** SF-36 subscales and global score for quality of life ^1^.

	Low-Gluten (3.5–4 g/day, *n* = 8)			Mid-Gluten (6.7–8 g/day, *n* = 6)			High-Gluten (10–13 g/day, *n* = 8)			
	Baseline	7-Day	*p*-Value ^†^	Baseline	14-Day	*p*-Value ^†^	Baseline	21-Day	*p*-Value ^†^	*p*-Value ^‡^
Physical functioning	100.0 (5.0)	95.0 (10.0)	0.10	100.0 (20.0)	82.5 (45.0)	0.17	100.0 (2.5)	100.0 (2.5)	0.94	0.13
Role physical	100.0 (25.0)	50.0 (37.5)	0.03	87.5 (25.0)	100.0 (50.0)	0.72	87.5 (37.5)	87.5 (62.5)	0.65	0.19
Role emotional	79.0 (26.0)	41.0 (31.0)	0.004	79.0 (26.0)	62.5 (32.0)	0.92	84.0 (27.5)	67.5 (40.5)	0.83	0.01
Bodily pain	76.0 (22.5)	53.5 (47.5)	0.008	76.0 (22.5)	64.0 (11.0)	0.87	66.5 (50.0)	69.5 (44.0)	0.30	0.15
Mental health	65.0 (20)	47.5 (17.5)	0.12	65.0 (20.0)	55.0 (20.0)	0.74	72.5 (15.0)	57.5 (37.5)	0.79	0.48
Vitality	87.0 (12.5)	56.0 (18.5)	0.02	87.0 (12.5)	62.5 (38.0)	0.50	100.0 (31.5)	68.5 (44.0)	0.34	0.67
Social interaction	100.0 (17.0)	33.0 (33.0)	0.007	100.0 (67.0)	100.0 (34.0)	0.10	100.0 (33.5)	100.0 (17.0)	0.39	0.30
General health	82.0 (18.0)	62.0 (34.0)	0.15	76.0 (20.0)	72.0 (16.0)	0.46	78.0 (10.0)	72.0 (24.0)	0.99	0.49

^1^ Data are shown as median values (interquartile range); ^†^ *p*-value for comparison within each group (low-, mid- and high-gluten) using a non-parametric Wilcoxon’s rank sum test; ^‡^ *p*-value for between-groups comparison (low-, mid-, and high-gluten) post diet using the Kruskal-Wallis test.

**Table 5:** Visual analogue scale scores for gastrointestinal symptoms ^1^.

	Low-Gluten (3.5–4 g/day, *n* = 8)			Mid-Gluten (6.7–8 g/day, *n* = 6)			High-Gluten (10–13 g/day, *n* = 8)			
	Baseline	7-Day	*p*-Value ^†^	Baseline	14-Day	*p*-Value ^†^	Baseline	21-Day	*p*-Value ^†^	*p*-Value ^‡^
Abdominal pain	0 (1.3)	2.6 (6.1)	0.16	5.3 (4.5)	4.9 (3.6)	0.87	0 (0.4)	0.5 (7.8)	0.22	0.66
Stool satisfaction	3.8 (9.1)	0 (3.4)	0.16	7.0 (9.8)	3.2 (5.4)	0.25	7.6 (8.8)	4.1 (8.8)	0.99	0.21
Abdominal bloating	0 (3.9)	3.0 (8.4)	0.19	5.4 (5.5)	5.2 (6.0)	0.87	0 (2.2)	3.5 (8.8)	0.19	0.68
Postprandial fullness	0 (4.4)	4.3 (7.4)	0.12	0 (6.7)	0 (2.9)	0.84	0 (0.0)	2.5 (6.6)	0.29	0.37
Early satiety feeling	0.2 (1.8)	3.5 (5.2)	0.13	0 (1.0)	0 (1.4)	0.84	0 (0.0)	1.3 (6.4)	0.09	0.36
Epigastric burn	0 (0.9)	2.0 (8.2)	0.07	0 (2.0)	0 (4.4)	0.85	0 (1.9)	0.7 (5.8)	0.41	0.42
General well-being	9.1 (2.9)	2.1 (2.9)	0.01	7.9 (3.1)	5.9 (4.5)	0.14	8.5 (1.9)	5.1 (4.3)	0.21	0.05

^1^ Data are shown as median values (interquartile range); ^†^ *p*-value for comparison within each group (low-, mid- and high-gluten) using a non-parametric Wilcoxon’s rank sum test. ^‡^ *p*-value for between-groups comparison (low-, mid- and high-gluten) post diet using the Kruskal-Wallis test.
